# Integrating Formative and Summative Clinical Skills Examinations to Promote Learning for Early Medical Students: A Mixed Methods Study

**DOI:** 10.1007/s40670-024-02086-w

**Published:** 2024-06-08

**Authors:** Gabrielle R. Goldberg, Doreen M. Olvet, Elizabeth K. Fiorino, Janice T. John

**Affiliations:** 1 Northwell, New Hyde Park, NY USA; 2 Department of Science Education, Zucker School of Medicine at Hofstra/Northwell, Hempstead, NY USA; 3 Department of Pediatrics, Albert Einstein College of Medicine, Bronx, NY USA

**Keywords:** Formative, Clinical skills, Assessment, Immediate feedback, Medical students

## Abstract

**Background:**

Medical educators aim to train students with high-quality clinical skills through the promotion of self-regulated learning and the development of lifelong learning skills. Formative clinical skills examinations (FCSE) allow for real-time learner-centered feedback and coaching which are key in promoting the development of expertise in early learners. This study assessed the impact of the integration of FCSE with learner-centered, real-time feedback and coaching based on an “educational plan-do-study-act” (PDSA) cycle on early medical students’ experience and performance.

**Methods:**

A mixed methods study was designed to assess the integration of FCSE for first- and second-year medical students. FCSE consisted of linked stations: students gathered a history from a standardized patient (SP), performed a hypothesis-driven physical examination followed by real-time learner-centered feedback and coaching. Each student met with one faculty and one SP to reflect on their performance, identify areas for improvement, re-practice of skills, and identify a plan for ongoing practice improvement. Students were surveyed upon completion of formative and summative clinical skills examinations. Student communication and clinical reasoning performance were compared to historical controls.

**Results:**

Students reported that FSCE improved the learning environment and helped prepare them for subsequent summative clinical skills examinations. Students appreciated the opportunity for practice and real-time feedback and reported applying their take-home points on subsequent exams. Student longitudinal performance was not impacted by the transition to FSCE.

**Conclusion:**

While labor-intensive, FCSE with real-time feedback and coaching are an effective means of promoting learning and should be considered for integration early in medical school curricula.

## Introduction

Medical educators have a responsibility to ensure medical students’ competence in the clinical skills needed to provide high-quality, compassionate, and safe patient care. Future clinicians need to apply medical knowledge and skills in the care of patients and develop the ability to reflect on their skills for ongoing practice improvement. Self-regulated learning (SRL) theory has been increasingly applied in medical education as it helps to support adult learners’ development of lifelong learning skills. SRL requires learners to plan, practice, and reflect on performance and SRL has been associated with academic success in the clinical environment [1, 2]. Formative assessment has been a means by which faculty promote SRL [3].

Formative assessment has been labeled a “key to deep learning”—learning for which students develop understanding, create meaning, and make connections to prior learning [4, 5]. As compared to summative assessment, aimed at determining a learner’s competence (assessment of learning), formative assessment aims to identify a learner’s skill level, areas for improvement, and plan for continued growth (assessment for learning) [6, 7]. The Liaison Committee for Medical Education (LCME) requires that medical schools develop assessment systems that include both formative and summative components [8]. Konopasek and colleagues propose building a formative assessment system beginning at matriculation and extending throughout the medical school curriculum [9]. Their proposal includes the framework of an “educational plan-do-study-act” (PDSA) cycle with underpinnings in theories of self-regulated learning and deliberate practice. The educational PDSA cycle involves learner-centered identification of areas for growth, active reflection, and opportunities for application of learning and is intended for collaboration between learners and teachers to guide learning across the medical education continuum [1, 10].

Clinical skills, traditionally developed later in training, are now being integrated earlier with the expectation that students will be prepared to participate as members of the clinical team when they begin clerkships[11, 12]. The objective structured clinical examination (OSCE) is used internationally to assess clinical skills competency. In the United States, the Step 2 Clinical Skills (CS), OSCE examination was developed to ensure minimum competencies in clinical skills for medical students [13]. The exam was praised for bringing increased attention to and investment in clinical skills programming across the country [14]. Many schools employed summative, high-stakes OSCEs to prepare students for Step 2 CS, and as a result, there was an increase in learner stress [15, 16]. Increasing costs and logistical challenges posed by the COVID-19 pandemic led to the discontinuation of Step 2 CS in January 2021 [17]. In response, medical education leaders have called for alternative considerations including the increased use of formative clinical skills examinations [18–22].

There is limited data supporting the use of formative assessment in clinical skills assessment. Formative OSCEs have been reported to increase learner self-confidence [23–25]. Formative feedback provided 1–2 weeks after an OSCE, while well received by students, resulted in improved performance only on identical summative OSCE stations [26]. In another study, the availability of formative feedback in the form of review of checklists and video recordings led to only half of students making use of this data [27]. Formative feedback in these studies was non-learner centered, not timely, and not generalizable to other contexts. There is no standard approach to the format of formative OSCEs, and none of the published studies are structured based on the PDSA framework.

**Fig. 1:**
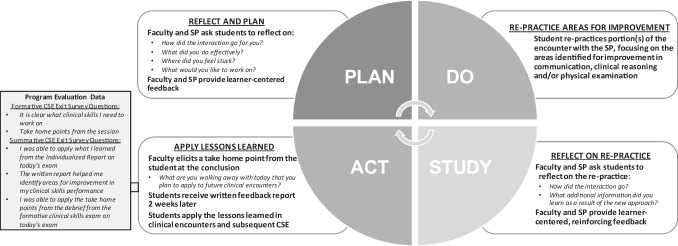
The educational PDSA cycle: formative clinical skills examination learner-centered feedback and coaching. SP, standardized patient; CSE, clinical skills examination

We used the opportunity of the discontinuation of Step 2 CS to reintegrate formative clinical skills examinations (FCSE) with real-time faculty and standardized patient (SP) coaching and feedback for first- and second-year medical students. Considering the educational PDSA framework and theories of self-regulated learning and deliberate practice, our FCSE provided learners with opportunities for application of clinical knowledge and skills, faculty-guided, learner-centered reflection on performance, real-time re-practice, and identification of a plan for ongoing practice improvement (Fig. 1).

In this study, we sought to determine if providing students with FCSE with real-time feedback and coaching based on the PDSA framework impacts student experience and performance. We aimed to assess student perception of the FCSE, students’ take-home points from FCSE, and how they were able to apply lessons learned from the FCSE to subsequent CSE based on student perception and performance data.

## Materials and Methods

### Setting

The study was conducted at the Zucker School of Medicine at Hofstra/Northwell (ZSOM). The ZSOM employs self-directed learning with a case-based/problem-based pedagogy with early clinical experiences. The curriculum introduces students to core clinical skills (communication skills for gathering a complete history and physical examination skills) during their first 7-week course. During the subsequent course, students are introduced to clinical reasoning during curricular sessions in which small groups of students work together to gather a patient history, generate diagnostic hypotheses, and plan a hypothesis-driven physical examination to test their diagnostic hypotheses. These sessions occur three times during each of the three remaining first-year (MS1) courses and three second-year (MS2) courses. All students complete a clinical skills examination (CSE) at the end of each of the MS1 and MS2 courses. The CSEs are conducted at the Clinical Skills Center at the Northwell Health Center for Learning Innovation (CLI). The Clinical Skills Center at CLI consists of 14 rooms designed to resemble outpatient examination rooms. CLI has a regular pool of previously screened SPs trained to the case and to the use of checklists to assess student performance. As part of standard operating procedures, SP educators conduct annual video reviews of CSE encounters to ensure reliability of checklists by course, case, and SP.

### Curricular Context

The MS1 year consists of four integrated courses: From the Person to the Professional: Challenges, Privileges, and Responsibilities (CPR), The Biologic Imperative (BI), Fueling the Body (FTB), and Continuity and Change: Homeostasis (HOM). The MS2 year consists of three integrated courses: Interacting with the Environment (IE), Host-Microbe Interaction (HMI), and The Human Condition (HC).

For the academic years spanning 2016 to 2021 (i.e., the classes of 2020 through the first year of the class of 2024), all end-of-course CSE, except for CPR, were conducted as summative examinations to prepare students for Step 2 CS. At the end of the CPR course, students completed an end of course single station formative assessment during which they practiced their communication and physical examination skills. The summative CSE resulted in an end of course grade of pass or fail and did not include any feedback or coaching. During the academic year 2021–2022, we introduced FCSE to two additional courses (BI and FTB) in the MS1 year and to the first course in the MS2 year (IE).

### Participants

First- and second-year medical students in the Class of 2024 (C24) and the Class of 2025 (C25) participated in the FCSE during the academic years 2021–2022 and 2022–2023 as part of standard end-of-course assessments (Table 1). C24 was introduced to FCSE in their MS2 year. C25 was introduced to FCSE in their MS1 year. The Class of 2022 (C22) completed all CSE prior to this intervention and served as a historical control. Due to the COVID-19 pandemic, several CSE for the class of 2023 were run on a virtual platform so could not serve as historical controls for the majority of the exams; C23 data was included as historical control for the one examination for which comparable C22 data was not available.

**Table 1:** Courses and clinical skills examination formats

	Course +	Class of 2022 *(Historical Control)*	Class of 2023 *(Historical Control)*	Class of 2024	Class of 2025
MS1 YEAR	CPR	Formative 1-Station	Formative 1-Station	Formative 1-Station	Formative 1-Station
	BI	Summative	Summative	Summative**	FORMATIVE++
	FTB	Summative++	Summative**	Summative++	FORMATIVE++
	HOM	Summative++	Summative**	Summative++	Summative++
MS2 YEAR	IE	Summative*	Summative++	FORMATIVE++	FORMATIVE++
	HMI	Summative++	Summative**	Summative++	Summative++
	HC	Summative**	Summative	Summative	Summative

+ *CPR* Challenges, Privileges and Responsibilities, *BI* The Biologic Imperative, *FTB* Fueling the Body, *HOM* Homeostasis, *IE* Interacting with the Environment, *HMI* Host-Microbe Interaction, *HC* The Human Condition

A full description of each course is available at https://medicine.hofstra.edu/education/md/fow/

Intervention noted with capitalization

++Course outcomes included in this study

^*^Different clinical case, excluded from analysis

^**^VIRTUAL exam/altered format, excluded from analysis

We included performance data only from students who previously consented at matriculation.

### Educational Intervention

**Fig. 2:**
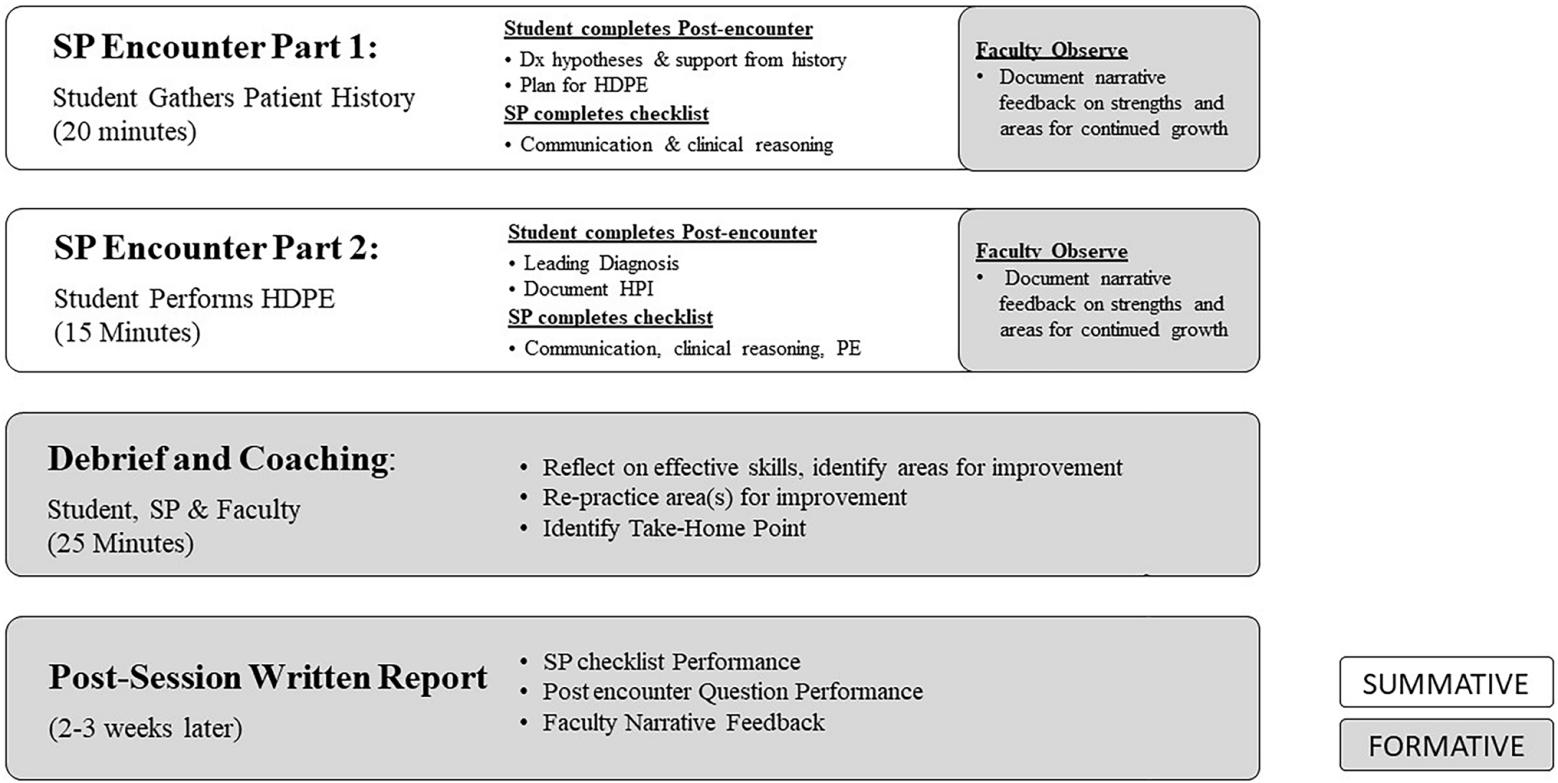
Linked station format. White boxes represent the format of summative clinical skills exams. Grey boxes indicate elements added for formative clinical skills exams. SP, standardized patient; HDPE, hypothesis-driven physical examination; HPI, history of present illness

The FCSE consisted of a “linked encounter” followed by real-time learner-centered feedback and coaching with a trained SP and clinical skills faculty member with built-in opportunity to re-practice identified areas for improvement. The linked encounters (Fig. 2), employed on all CSE since 2016, were developed to assess students’ communication, physical diagnosis, and clinical reasoning skills. During the first part of the linked encounter, students gathered a history from an SP and completed a post-encounter to assess their diagnostic hypotheses and their plan for the hypothesis-driven physical examination. During the second part, students continued the encounter with the same SP during which they conducted the hypothesis-driven physical examination. The linked encounter concluded with post-encounter documentation of the patient’s leading diagnosis and history of present illness. During the FCSE, faculty observed the linked encounter from a computer lab and documented observations on what the student did effectively and opportunities for practice improvement. The FCSE concluded with a 25-min individualized, learner-centered debrief with feedback and coaching facilitated by the faculty and SP.

We developed a protocol for a student-SP-faculty triad learner-centered feedback and coaching that employed elements of a PDSA cycle including principles of psychological safety, self-regulated learning theory, and deliberate practice [1, 10, 28] (Fig. 1). The protocol established psychological safety through introductions, check-in on the student’s emotional state, and scripting a transparent plan for the process. After establishing safety, learners were asked to reflect on the encounter to identify their strengths and areas for improvement or “learning edge” and worked with faculty to brainstorm a plan for improvement (PLAN). Learners re-practiced the skill identified as their learning edge (DO). Students reflected on the re-practice with reinforcing feedback from the faculty and SP (STUDY). At the end of the feedback session, learners reflected on the process and identified an action plan for ongoing practice improvement (ACT).

Students’ clinical skills performance during the encounters is assessed on SPs checklists completed after each encounter. Clinical skills faculty grade all student post-encounter questions anonymously using a grading rubric. About 2 weeks after the FCSE, learners receive an individualized feedback report including performance data from the SP checklists, faculty graded post-encounters, and faculty narrative feedback.

### Clinical Skills Faculty

Faculty were recruited from a pool of ZSOM faculty. Nineteen faculty participated in the FCSE during the academic years 2021–2022 and 2022–2023. A faculty development curriculum was developed to prepare faculty with the knowledge, skills, and attitudes needed to apply a uniform, learner-centered approach to our FCSE feedback and coaching model. Faculty attended a 90-min interactive, skills-based faculty development session before each FCSE.

### Outcome Measures

To assess student perception and experience, students were invited to complete a brief, voluntary, anonymous paper exit survey. Students were given the paper exit survey immediately after completion of the CSE, and surveys were collected in real-time.

#### FCSE Exit Survey

C24 completed the FCSE exit survey after their first FCSE. This survey consisted of one 4-point Likert-style question (strongly disagree, disagree, agree, strongly agree) which asked them to reflect on if it was clear what clinical skills they need to work on and two open-ended questions eliciting their personal take home point and additional feedback.

#### Summative CSE Exit Survey

C25 students completed the summative CSE survey after completion of their first summative CSE in the MS1 year. Both C24 and C25 completed this exit survey after the first summative CSE in the MS2 year. The summative exit surveys consisted of three, 4-point Likert-style (strongly disagree, disagree, agree, strongly agree) and one open-ended question. The Likert questions asked students to reflect on whether they were able to apply their take-home points and lessons learned from the individualized written report from the prior FCSE to that day’s summative CSE. The final open-ended question asked for additional thoughts about the clinical skills assessment.

#### CSE Performance

To assess student performance on CSE, we used student performance on SP checklists assessing communication and clinical reasoning. The communication checklists, based on the ZSOM core communication curriculum, included 20 items that were consistent across all CSE. The clinical reasoning checklists were case-specific based on the chief concern for the patient and included 16–18 items assessing the data gathered during both history and hypothesis-driven physical examination. The number of checklist items assessing physical examination techniques varied between courses and were therefore not included in our analysis. The pandemic impacted our exam administration; data gathered from CSE run in a virtual format were excluded from the study. We therefore analyzed data from 4 of the 7 clinical skills examinations (Table 1).

The Hofstra University Institutional Review Board deemed this project exempt from full review.

### Statistical Analysis

Data was statistically evaluated using IBM SPSS Statistics (SPSS Inc., Chicago, IL, USA, Version 28.0). Descriptive statistics are presented as the frequency and percent of agree and strongly agree for the exit survey questions (4-point Likert scale) and as the percent correct for the SP communication and clinical reasoning checklist performance. Mann–Whitney *U* tests were used to evaluate group differences (C24 vs. C25) in the responses on the summative CSE exit surveys completed in the MS2 year. Analysis of variance (ANOVA) was used to determine group differences on communication and clinical reasoning checklist performance. Post hoc independent sample *t*-tests were performed for follow-up for significant findings. A *p* value ≤ 0.05 was considered statistically significant except for post hoc *t*-tests for which we applied Bonferroni correction requiring a *p*-value < 0.016 for significance.

### Qualitative Analysis of Narrative Responses on Exit Surveys

A thematic analysis was conducted on the narrative responses to exit surveys. Two of the researchers (GG and EF) used an iterative and inductive approach to familiarize themselves with the data, generate initial codes, search for themes, and define and name the themes [29]. Responses to the initial exit survey were reviewed independently to generate initial codes, which were applied to each subsequent survey with clarification of wording and addition of codes. This process allowed for new codes to be added until theoretical saturation was reached. The researchers then met to reach consensus on final codes. After consensus was reached, the two researchers independently coded all responses and then met to reach a final consensus on coding of individual responses and identification of themes. A third researcher (JJ) was available to review any codes or responses which lacked initial agreement. Independent coding of survey data enabled assurance of trustworthiness and reflexivity [30, 31]. The researchers maintained code books to capture impressions and reflections on the coding process and note any potential biases.

## Results

All students in C22, C23, C24, and C25 completed the CSEs as part of our standard educational protocol. C24 and C25 completed voluntary exit surveys at 2 points in time. C24 completed the formative CSE exit surveys after their first FCSE (*N* = 103, 100% response rate) and completed the summative CSE exit survey after the subsequent summative CSE which took place approximately 2 months later (*N* = 98, 95% response rate). C25 completed the summative CSE exit surveys after the first summative CSE in both the MS1 year (*N* = 97, 96% response rate) and MS2 year (*N* = 98, 97% response rate).

### Formative CSE Exit Surveys

The overwhelming majority of respondents (99%) agreed or strongly agreed with the statement “It is clear to me what clinical skills I need to work on.” Students were asked to share their take home points, and the following themes were identified, in order of frequency: communication (general history taking, empathy/connection, importance of review of systems), clinical reasoning (general clinical reasoning, importance of diagnostic time out, avoiding early closure), physical examination (technique and hypothesis-driven physical examination), preparation and organization (content preparation and organization during encounter), appreciation (opportunity for practice and real-time feedback), and learning environment (less anxiety, less stress, and built confidence).

### Summative CSE Exit Surveys

The summative CSE exit survey was completed by C25 in both the MS1 (HOM) and MS2 (HMI) years and by C24 in the MS2 year (HMI).

On the survey completed after their first summative CSE in their MS1 year, the majority of C25 reported that the written report, received after the prior FCSEs, and helped to identify areas for improvement, and 92.8% of C25 students agreed or strongly agreed with the statement “The Formative Clinical Skills Individualized report helped me identify areas for improvement in my clinical skills performance.” The majority of students in C25 reported they were able to apply lessons learned from the prior FCSE to their first summative CSE: 96.9% of C25 students agreed or strongly agreed with the statement “I was able to apply the take home points during course 2 and course 3 Formative Clinical Skills Exam to today’s exam” and 93.8% of C25 agreed or strongly with the statement. “I was able to apply what I learned from the individualized report to today’s exam.”

On the survey completed after the first summative exam in the MS2 year, the majority of students in both C24 and C25 reported that the written report received after the prior summative CSE helps to identify areas for improvement, and 90% of C24 and 84% of C25 agreed or strongly agreed with the statement “The Formative Clinical Skills Individualized report helped me identify areas for improvement in my clinical skills performance.” There was a significant difference between the groups (*U* = 4058, *p* = 0.035). C24, who experienced only one FCSE, were more likely to report that the written report helped them to identify areas for improvement.

Students in both classes reported that they were able to apply lessons learned from the prior FCSE to that day’s summative exam. The majority of students were able to apply their take home points from the prior exam: 91.8% of students in C24 and 94.7% in C25 agreed or strongly agreed with the statement “I was able to apply the take home points during the Formative Clinical Skills Exam to today’s exam.” There was no significant difference between the groups (*U* = 4541, *p* = 0.63). The majority were also able to apply feedback from the written report: 88.5% of students in C24 and 90.7% in C25 agreed or strongly agreed with the statement “I was able to apply what I learned from the individualized report to today’s exam.” There was no significant difference between the groups (*U* = 4359, *p* = 0.36).

### Narrative Feedback on Formative and Summative Exit Surveys

All exit surveys asked students to share narrative feedback on the clinical skills assessments. The following themes were identified on qualitative analysis (Table 2):

**Table 2:** Thematic analysis of feedback from exit surveys

Themes (frequency)	Codes (frequency)	Representative comments
Programmatic feedback (127)	Suggestions for improvement/logistical feedback (54)	*I personally think it’d be better to not have the clinical skills exam the day before our essay exams, Since it makes it a little more difficult to prepare for our essay exams… (C25 HMI)*
	Continue formative feedback, include feedback even with summative (41)	*…more feedback. A chance to try again. Make all of them formative please:) (C24 HMI)*
	SP/faculty feedback (16)	*… I really enjoyed the post encounter feedback from the faculty. Made this a learning session as opposed to an assessment. (C24 IE FCSE)*
	Course-specific feedback (16)	*I think we would benefit from more PPS (Patient, Physician, and Society) clinical skills sessions—such as motivational interviewing and action planning (C25 HMI)*
	Area for improvement in faculty feedback (2)	
Receiving feedback (54)	Formative feedback is helpful (27)	*I thought this formative clinical skills session was very helpful (C24 IE)*
	Real-time feedback (17)	*I really appreciated the chance to have real-time feedback during the clinical skills exam–—they were so helpful to identify even the most minor (but still important) opportunities for growth. The real time aspect was really key b/c it forced me to very thoughtfully reflect and gave me a chance to practice & repeat… (C25 Hom)*
	Written report is helpful (8)	*the formative was a long time ago but the report was nice to see for specific feedback in writing (C24 HMI)*
Feelings (44)	More comfortable/less stress on formative vs summative (27)	*I definitely felt more stressed with this than the formative exam & wish we had the 1-on-1 debrief opportunity again with this course. (C24 HMI)*
	Gratitude (27)	*Thank you for the feedback session at the end. Please continue providing student immediate feedback post exams even if the exam is summative. (C24 IE)*
Learning environment (20)	Better learning environment with formative (20)	*Without the pressure of the grade, I felt I could focus more on my interactions and be really present to the experience. I felt more real and through the comfort that provided me, I felt better able to remember information as well. (C24 IE)*
Preparation (16)	Applied feedback from formative to summative (8)	*I would love to have some feedback on my performance today, considering I felt like I applied my feedback to my performance today. (C25 HMI)*
	Preparation no different for formative than summative (4)	*I did not feel that my level of preparation or performance was lessened by the formative nature of the exam. If anything, I performed better and was able to focus on the learning. (C24 IE)*
	Felt prepared (4)	*Having the faculty feedback session during [past FCSE] were very helpful in preparing for today (C25 HOM)*
	Did not feel prepared (2)	

#### Programmatic Feedback

Students shared suggestions for feedback on administration of clinical skills examinations, including timing relative to their other examinations. C25 was more likely to share this type of logistical feedback*.* Many students requested continuation of formative feedback including the suggestion that real-time feedback also be offered during summative CSE.

#### Receiving Feedback

Students shared that formative feedback was helpful. Students specifically highlighted the importance of real-time feedback and the availability of the written report.

#### Emotions

Students commented that they experienced less stress associated with FCSE as compared to summative CSE. Students also expressed feelings of gratitude and appreciation about the addition of FCSE and availability of real-time feedback.

#### Learning Environment

C24 were more likely to comment that the environment during FCSE was more conducive to learning than during summative CSE. Students attributed this to decreased anxiety and availability of learner-centered real-time feedback from faculty and SPs.

#### Preparation

Students did not report that they prepared differently for FCSE and summative CSE. They also reported that the FCSEs helped them prepare for subsequent summative CSE as they believed they were able to apply feedback from the FCSE during the summative CSE.

### Student Performance

Student performance on SP communication and clinical reasoning checklist items were calculated as a percentage of full credit. Performance data for historical control (C22, *N* = 98; C23, *N* = 100) and intervention group (C24 *N* = 99; C 25, *N* = 98) was analyzed for two MS1 CSE (FTB and HOM) and two MS2 CSE (IE and HMI) (Table 3).

**Table 3:** SP checklist performance by course

Course	Group	N	Clinical Reasoning		Communication Checklist	
			Mean (SD)	ANOVA	Mean (SD)	ANOVA
FTB	C22	98	80.2 (12.2)	F(2,293) = 2.4, p > 0.05	91.8 (6.1)	F(2,292) = 1.7, p > 0.05
	C24	99	77.3 (10.4)		94.0 (7.6)	
	C25+	98	77.1 (10.7)+		91.4 (6.3)+	
HOM	C22	98	85.5 (9.5)	F(2,292) = 15.6, p < 0.001*	93.8 (6.5)	F(2,292) = 1.3, p > 0.05
	C24	99	83.8 (7.7)		94.3 (5.5)	
	C25	98	78.8 (9.1)		93.1 (4.9)	
IE	C23	100	85.2 (8.6)	F(2,294) = 16.3, p < 0.001*	90.4 (4.2)	F(2,296) = 24.8, p < 0.001*
	C24+	99	77.5 (11.3)+		94.0 (5.7)+	
	C25+	98	78.3 (11.1)+		93.6 (4.9)+	
HMI	C22	98	80.6 (9.4)	F(2,292) = 0.7, p > 0.05	95.4 (4.7)	F(2,292) = 0.2, p > 0.05
	C24	99	79.1 (8.2)		95.4 (4.4)	
	C25	98	80.2 (9.7)		95.7 (4.5)	

*FTB* Fueling the Body, *HOM* Homeostasis, *IE* Interacting with the Environment, *HMI* Host-Microbe Interaction

^*^On post hoc t-test: HOM clinical reasoning C25 < C22 and C24; IE clinical reasoning C23 > C24 and C25; IE communication: C23 < C24 and C25

+Indicate formative examinations

#### Clinical Reasoning

**Fig. 3:**
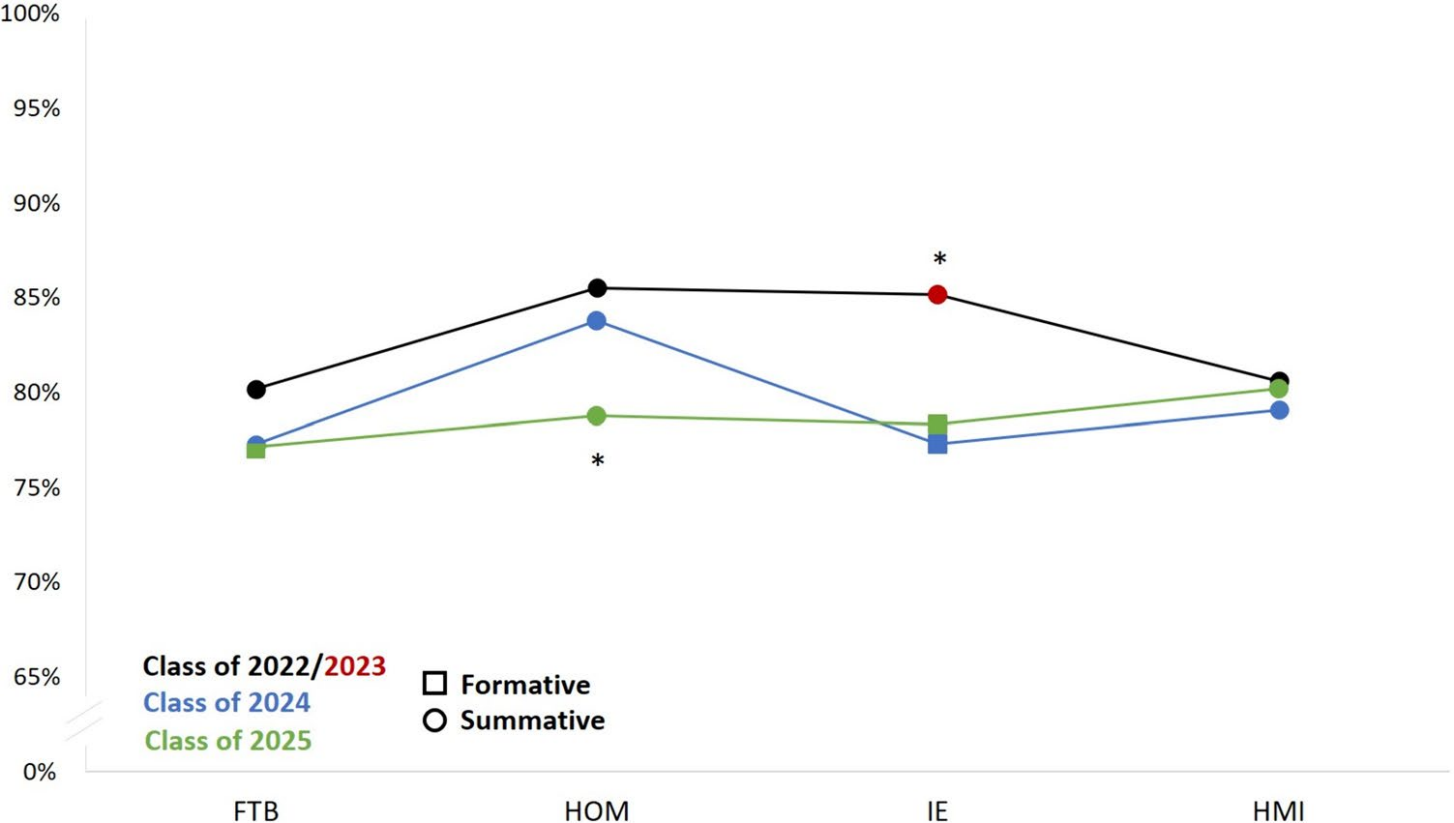
Student performance on clinical reasoning checklist. * indicates *p* < 0.001

**Fig. 4:**
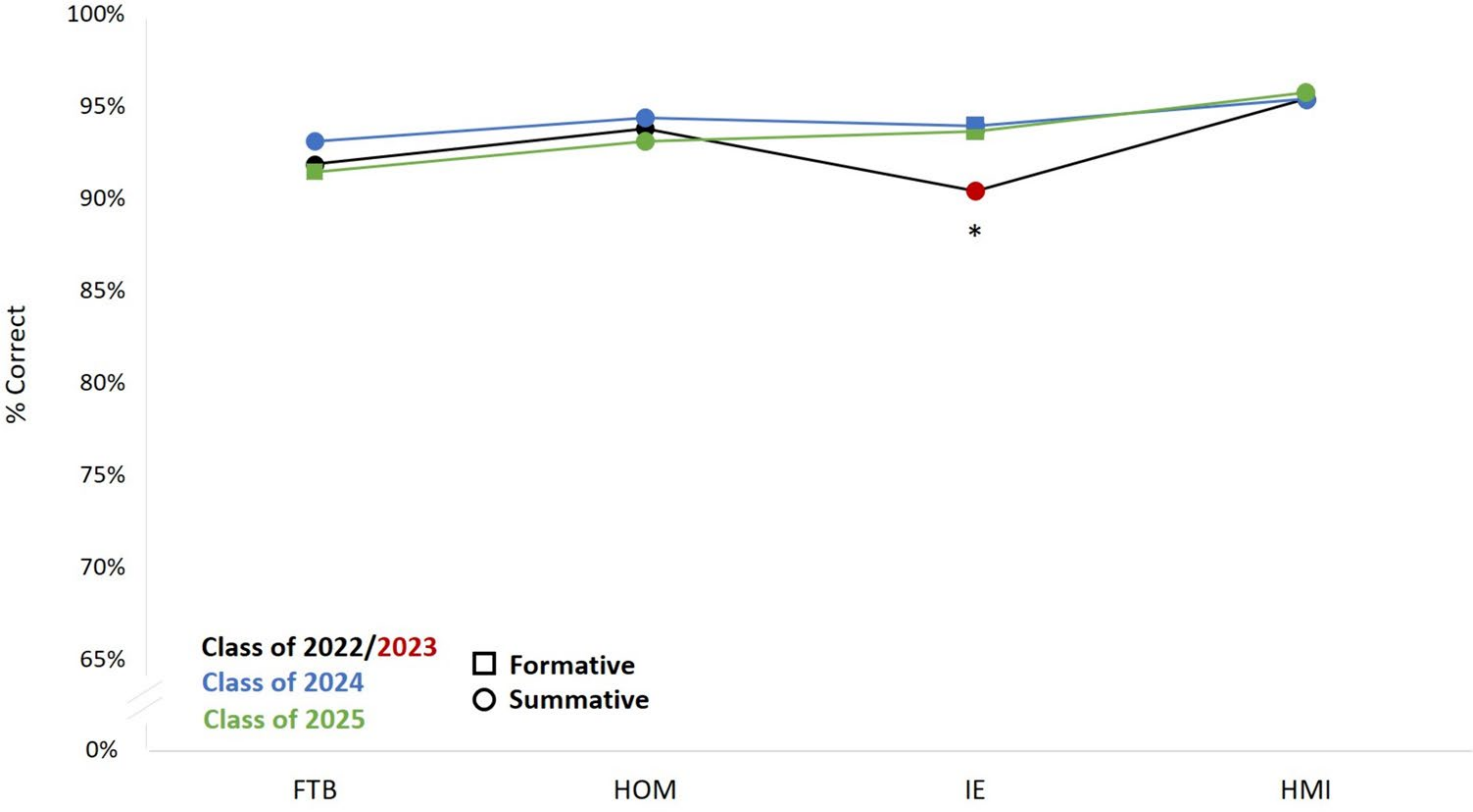
Student performance on communication checklist. * indicates *p* < 0.001

There was a significant difference in the clinical reasoning checklist performance between the groups on the HOM exam and on the IE exam (Fig. 3). There was no difference between groups on the FTB or HMI exams (Fig. 4).

C25 performed significantly lower in the clinical reasoning domain on their first summative CSE (HOM) than both C24 (*t*(195) = 4.19, *p* < 0.001) and C22 (*t*(194) = 5.09, *p* < 0.001). There was no difference in clinical reasoning checklist performance between C22 and C24 on the HOM exam which both classes completed as a summative exam.

Conversely, the historical control cohort C23, who completed the IE exam as a summative exam, performed significantly higher on clinical reasoning scores than C24 (*t*(195) = 4.19, *p* < 0.001) and C25 (*t*(195) =  − 5.91, *p* < 0.001), for whom the IE exam was formative. There was no difference in clinical reasoning scores between C24 and C25 on the IE exam.

#### Communication

There was a significant difference in the communication checklist performance between the groups only on the IE exam. C23, performed lower than both C24 (*t*(197) = 4.95, *p* < 0.001) and C25 (*t*(196) = 4.98, *p* < 0.001) on the communication checklist. C23 CSE were significantly more impacted by the COVID pandemic than other classes. C23 completed their other three CSE in a virtual format which differed from the other classes which likely impacted their communication checklist performance.

## Discussion

The discontinuation of the national high-stakes clinical skills examination created opportunities for innovation. The aim of this study was to evaluate FCSE with real-time coaching based on the educational PDSA framework and to assess the impact on performance and experience of medical students early in their training. The intention of using the PDSA framework was to promote the development of self-regulation as our learners develop their clinical skills. The exit surveys encouraged learners to reflect on their take-home points after the FCSE and how to apply these points as well as written feedback to subsequent clinical skills exams. Students walked away from the FCSE with clearly identified strengths and areas for improvement that included specific clinical skills and the importance of preparation and organization during clinical encounters. Students reported that they could apply lessons learned from this feedback to their subsequent summative clinical skills exams.

The FCSE were well received by students. Students expressed gratitude for the chance to participate in the FCSE, found formative feedback helpful, and highlighted the importance of real-time feedback from faculty and SPs. Notably, the theme of learning environment came up both in the formative and summative CSE exit surveys. There has been increased attention to the importance of optimizing the learning environment in medical education [32]. However, there is limited data to support what interventions lead to improvements in the learning environment [33]. Interventions described in the literature include programs that establish positive interpersonal relationships, support learner autonomy through impacts on personal, social, physical structure, and organizational levels. One program designed to improve the learning environment by implementation of a pass/fail system to lower the stakes in grading was associated with improvements in learner well-being [34]. Multiple organizations have called for the development of interventions to advance medical student well-being, particularly during and after the COVID-19 pandemic [8, 35]. Higher levels of learner stress were anecdotally observed by faculty at our institution after we shifted all clinical skills examinations to summative. Students in our study noted feeling more comfortable and less stressed with FCSE compared to summative CSE. Future work should be designed to rigorously explore the relationship between learning environment, student emotional well-being, and assessment and grading systems.

While the majority of C25 reported that they were able to apply the take-home points from FCSE to subsequent summative CSE, their performance on the clinical reasoning checklist on the subsequent summative CSE was lower than that of the C22 and C24, both of whom completed the exam as summative. There are several potential explanations for the lower performance on C25’s first summative CSE. One potential explanation worth noting is that during the summative CSE, students completed the linked stations as two stations out of a four station CSE. The additional cognitive load of the first exposure to two extra stations and anxiety related to the summative nature of the CSE, likely both played a role [36]. Presumably, prior experience completing summative CSE impacted performance for C22 and C24. All classes performed very well on the communication checklist; performance was not impacted by the format of the CSE. With the exception of one exam, C23 data could not be used as historical controls for the majority of the courses as most of their CSE were run in a virtual format. We hypothesize that fewer opportunities for in-person interactions with SP resulted in a negative impact on C23 student core communication skills performance.

Our study is limited as it occurred in a single institution with resources available to staff FCSE with clinical skills faculty and SPs. We recognize these resources may not be readily available across institutions. Our pool of historical controls was limited as CSE were administered virtually because of the pandemic. Given ongoing quality improvement initiatives that occur in all curricula, as well as unmeasured impact of the pandemic on learning, it is difficult to interpret how well the historical groups truly served as controls. Future efforts should include assessment of students’ longitudinal performance on clinical skills through the remainder of their medical school training, including evaluation at a higher Kirkpatrick level—the ultimate impact on patient care. The assessment of the impact of the FCSE on long-term learning outcomes is limited by ongoing curricular improvement initiatives and the impact of each is difficult to measure independently.

Students appreciated the opportunity to receive real-time, individualized feedback and coaching. While we have a cadre of trained clinical skills faculty, our staffing paradigm did not allow for consistent individualized pairings of faculty and students over time. Our program would be strengthened by creating a staffing model to ensure pairing of faculty and students to create longitudinal coaching relationships [37]. This longitudinal coaching relationship would align with Ottawa Consensus recommendations for competency assessments in clinical skills that provide evidence of “learning progress and readiness for practice” [38]. This would also further support the intention of the educational PDSA which conceptualizes formative assessment as a longitudinal process [9].

Summative end of course CSE at our institution were originally integrated to increase student buy-in, preparation, and performance. Studies have demonstrated that the introduction of summative CSEs shifted student preparation to focus on clinical skills learning [39]. Despite potential concerns, the transition to the FCSE did not negatively impact student preparation and performance on MS2 year summative CSE. Students expressed desire for continuation of FCSE and increased opportunities for formative feedback. Next steps will include integration of the PDSA cycle feedback and coaching into the summative CSE. This will allow for continued real-time formative feedback and maintain the motivation provided by summative examinations, while providing quantitative data to ensure minimum competence in learners before progressing in the continuum of medical education.

## Conclusions

Assessment drives learning, whether it is assessment of, or for that learning. The COVID-19 pandemic and the discontinuation of Step 2 CS stimulated educators to rethink clinical skills assessments. This study demonstrates that formative assessments designed with an educational PDSA framework allow learners to identify specific skills to practice and apply the lessons learned—key elements of self-directed, life-long learning. Formative assessments with real-time, individualized coaching and feedback can drive learning, while maximizing the learning environment and minimizing stress and anxiety in our learners. A balance of summative CSE and FCSE should be considered at all institutions.

## Data Availability

The datasets used and/or analyzed during the current study are available from the corresponding author on reasonable request.
